# Unveiling B7/CD28 family proteins in hepatocellular carcinoma: insights into communication and prognostic significance

**DOI:** 10.3389/fimmu.2025.1583597

**Published:** 2025-07-30

**Authors:** Yan Cai, Xiaodi Liu, Tao He, Yangming Xu, Shuxian Chen, Xianliang Tan, Shouchao Wei, Zimeng Wu, Liying Xiao, Baoming Luo

**Affiliations:** ^1^ Department of Ultrasound, Central People’s Hospital of Zhanjiang, Zhanjiang, China; ^2^ Department of Ultrasound, Sun Yat-sen Memorial Hospital, Sun Yat-sen University, Guangzhou, China; ^3^ Laboratory of Ultrasound Imaging and Drug, West China Hospital, Sichuan University, Chengdu, Sichuan, China; ^4^ Guangdong Provincial Key Laboratory of Malignant Tumor Epigenetics and Gene Regulation, Sun Yat-sen Memorial Hospital, Sun Yat-sen University, Guangzhou, China

**Keywords:** hepatocellular carcinoma (HCC), tumor immune environment, immunotherapy, B7/CD28 family members, inhibitory B7 family members, B7H3, single-cell RNA sequencing

## Abstract

**Background:**

Immunotherapy has made remarkable achievements in cancer treatment, but it still faces the challenge of limited response rates in liver cancer therapy. Although there has been extensive research on the role of programmed cell death-ligand 1 (PD-L1) in hepatocellular carcinoma (HCC), our understanding of the effects of other inhibitory B7/CD28 family members is still limited despite advancements in prognostic tools, more specific, accurate, and robust biomarkers are required to improve HCC patient prognoses.

**Methods:**

We acquired the single-cell sequencing data from relevant literature and selected 42 liver tissue samples, including 89,246 cells from HCC patients, to investigate the expression, localization, and intercellular communications of the B7/CD28 family in HCC. Within the Cancer Genome Atlas dataset, we utilized Lasso and Cox regression analyses to develop a risk model for identifying the most pertinent B7/CD28 family proteins associated with prognosis. Subsequently, we conducted a retrospective analysis of 94 HCC patients who underwent hepatectomy at our institution and determined the prognostic significance of this malignancy.

**Results:**

Based on the single-cell RNA sequencing data, we have delineated various members of the B7/CD28 family and their corresponding receptors. We have elucidated their distribution on tumor cells and immune cells, revealing active intercellular communications among tumor cells, fibroblasts, and epithelial cells. Through the implementation of Lasso, we have pinpointed a significant correlation between the B7H3 molecule and prognosis. Leveraging multiplex immunofluorescence, we were able to discern three distinct patterns of B7H3 expression—tumor-associated, stroma-associated, and a hybrid form encompassing both. Notably, the presence of B7H3 in the stroma exhibited the most robust correlation with prognosis. Furthermore, the efficacy of our prognostic signature was validated through clinicopathological analyses conducted at our institution.

**Conclusions:**

In conclusion, the B7/CD28 family plays an active role in the tumor immune microenvironment and cellular communication. B7H3 could serve as an indicator for predicting the outcome of HCC. Additional investigation is required to validate these discoveries in future groups of individuals and assess their viability as therapies guided by biomarkers.

## Graphical Abstract

Schematic diagram. Workflow of the present study.

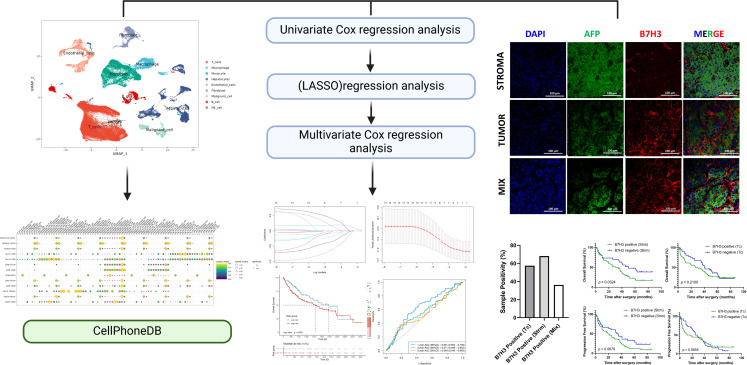

## Introduction

Hepatocellular carcinoma (HCC) is a significant contributor to the global cancer burden, affecting populations worldwide. HCC is a major cause of cancer-related fatalities globally (1). This highlights the pressing need for effective treatment approaches to enhance clinical outcomes.

Antibody blockade targeting inhibitory receptors has shown clinical efficacy by reversing T-cell exhaustion in various cancers (2). However, single-agent anti-PD1 or -PDL1 therapies have limited objective response rates of 15-25% in liver cancer (3). Combined blockade of other receptors, such as Tim3, can enhance therapeutic benefits (4).

The B7/CD28 family of molecules, comprising several immune checkpoint proteins and their receptors, such as CD28, CTLA4, PDCD1, etc. have gained considerable attention in the field of cancer therapy (5). These molecules, especially in combination therapies, have attracted attention due to their functional roles. The B7/CD28 family includes B7-1, B7-2, PD-L1, PD-L2, ICOSL, CD276, B7S1, VISTA, NCR3LG1, and HHLA2 (6). Various normal and tumor tissues express these molecules, which are crucial for preserving self-tolerance and promoting immunity against tumors (7). Currently, there is a deficiency in systematic studies exploring the precise molecular distribution of B7/CD28 family proteins within liver cancer, as well as the cellular interactions they mediate.

The advent of single-cell RNA sequencing (scRNA-seq) technology has provided a pivotal tool for unveiling the phenotypes and classification of immune cells within the tumor microenvironment (TME) (8). By detecting transcriptomic information within individual cells, this technique enables an in-depth examination of molecular expression levels across diverse immune cell populations (9). Consequently, it offers a novel biological approach to elucidating the distribution and interaction mechanisms of the B7/CD28 family and their receptors.

In this study, we analyzed the single-cell expression patterns of B7/CD28 family molecules in both healthy liver and HCC tissues with scRNA-seq and performed cell-cell communication analysis to explore information transmission that B7/CD28 family protein-mediated between cells.

We further utilized bioinformatics techniques to clarify the prognostic significance of B7-related compounds and their receptors. B7H3, also known as CD276, a member of the B7/CD28 family proteins, has been proven to possess the best diagnostic value for prognosis in the prediction model. Subsequently, immunohistochemistry (IHC) and multiplex immunofluorescence (IF) techniques were employed to evaluate the presence of B7H in tumor cells and the stroma (non - malignant cells, including fibroblasts, endothelial cells, macrophages, and other cells that provide a structural and functional support for tumor cells in the TME). We explored the relationship between the expressions of B7H3 and its associations with clinicopathological features. Finally, we investigated the impact of different expression patterns of B7H3 on the prognosis of HCC patients.

Our discoveries suggest that the B7/CD28 family exhibits dynamic cellular communication within the TME, wherein the expressions of B7H3 notably influence the prognosis of patients with HCC. The research emphasizes the predictive significance of B7/CD28 family proteins and their receptors in HCC, underscoring B7H3 expressed in the stroma as potential prognostic markers. This finding has the potential to assist in devising personalized therapeutic approaches for individuals with HCC (**Graphical Abstract**).

## Materials and method

### Single-cell RNA sequencing data processing

In our study, we obtained 18 hepatocellular carcinoma (HCC) samples with single-cell RNA sequencing (scRNA-seq) data and 24 normal liver samples from four public datasets: GSE136103 (10), GSE168933 (11), GSE125449 (12), and GSE149614 (13). These datasets were selected based on the availability of relevant data and compatibility with our research objectives. In the original studies corresponding to these datasets, the HCC samples were derived from patients who had been clinically diagnosed with HCC and subsequently underwent surgical resection. The healthy liver samples were resections taken from tissues adjacent to tumor metastasis sites in the liver of these surgical patients. We utilized the Seurat package (version 4.0) in R statistical software (version 4.0.5, The R Foundation for Statistical Computing, Vienna, Austria, URL https://www.R-project.org/) for downstream scRNA-seq analysis. Following data integration from the four datasets, we conducted quality control (QC), defining low-quality cells as those meeting the criteria: nFeature_RNA > 9000 & nFeature_RNA < 200, nCount_RNA > 40000 & nCount_RNA < 3. Usually, cells with a mitochondrial genome percentage exceeding 20% are filtered out to identify low-quality or apoptotic cells. The filtering standard for red blood cell related genes is greater than 5%.

### Data integration and the dimensionality reduction

After filtering out low-quality data, we proceeded with data normalization. We modeled the inherent mean-variance relationship in single-cell data through the FindVariableFeatures function (nfeatures = 2000), identifying a subset of features with significant variability between 89,246 cells. We integrated data from the four datasets to address batch effects using the FindIntegrationAnchors function and “IntegrateData” method. We reduced PCA dimensionality on the selected highly variable genes with the RunPCA function (npcs = 30). We utilized “FindNeighbors” and “FindCluster” (resolution = 0.5) for data clustering and partitioning. We employed RunUMAP and RunTSNE on the top 20 principal components based on the optimal dim value for non-linear dimensionality reduction.

### Cell-clustering and annotation

For differential analysis, we used the “FindAllMarkers” method to identify differentially expressed genes between clusters, filtering out genes with padjust < 0.05 for statistical significance. Finally, we visualized gene expression through “VlnPlot” and “DoHeatmap” functions, generating violin plots and heatmaps, respectively.

For cell type assignment, we utilized the SingleR software (Single-cell Recognition of cell types, version 2.0) and manually defined cell types based on the high-specificity expression of marker genes referenced in the literature.

We identified cell types in each subgroup using known lineage markers: T cells (CD3D, CD3E, CD3G), macrophages (CD163, CD68), monocytes (CCR2, S100A8), hepatocytes (ALB, HNF4A, CYP3A4), endothelial cells (CD31, CDH5, von Willebrand factor), fibroblasts (COL1A1, COL3A1, FAP), malignant cells (AFP, KRT19, EpCAM), B cells (CD19, CD79A, IgH), and NK cells (NKG7, CD56, perforin).

### Cell-cell communication analysis

CellPhoneDB software was used to identify ligand-receptor pairs among the major cell types between tumor cells and other cell populations in the TME, particularly the B7/CD28 family proteins. Using the “cpdb_statistical-analysis” method, pairwise comparisons were made for all cell types. By randomly arranging the cluster labels of all cells (default to 1000 times), the average receptor and ligand expression levels between different cell types were calculated. For each receptor ligand pair between cell types, a p-value was generated based on a zero distribution, which represents the strength of receptor ligand interactions.

### Bioinformatics analysis

The STAR-counts data and clinical information on liver cancer were obtained from the Cancer Genome Atlas (TCGA) database (https://portal.gdc.com). Subsequently, the TPM format data was extracted and normalized using the log_2_ (TPM+1) method. In the end, we kept samples that had RNAseq data and clinical information, and we utilized liver cancer samples for further analysis.

We examined the proteins of the B7/CD28 family and their corresponding receptors, including CD80, CD86, CD274, PDCD1LG2, ICOSLG, CD276, VTCN1, VSIR, HHLA2, PDCD1, ICOS, CTLA4, CD28, TREML2, NGFR. For this research, we employed the R software (R software, version 4.0.3) glmnet package to combine information on survival status, survival time, and gene expression data. We utilized the lasso-cox technique to conduct regression analysis. Previously, to conduct feature selection, we used the LASSO algorithm in the glmnet package of R software and carried out 10-fold cross-validation.

In KM survival analysis, the log-rank test was employed to assess the disparities in survival among various groups. To evaluate the accuracy of the predictive model, a time-dependent ROC analysis was conducted. Samples that had a follow-up period of fewer than 30 days or an incomplete survival status were not included in the analysis.

### Individuals and biological specimens

The Ethics Committee of Zhanjiang’s Central People’s Hospital (PJ[IIT-2023-020-01]) approved this retrospective cohort study. This study included 94 patients with HCC consecutively at Zhanjiang Hospital over three years (from March 2015 to September 2019). HCC diagnosis was made for all patients using clinical, laboratory, and imaging findings. The patients underwent surgical resection for HCC, and informed consent was not available for this retrospective study. The present study excluded patients who underwent any interventional treatment. All hepatocellular carcinoma patients who met the inclusion criteria during the study period were enrolled.

Data from the medical records were gathered, including sex, age, lab outcomes, imaging discoveries, tumor category, and imaging staging. We collected Formalin-Fixed Paraffin-Embedded (FFPE) blocks from each patient from the pathology department of our center, which were fixed with formalin and embedded in paraffin. Sections of tissue embedded in paraffin were cut at a thickness of 4 μm.

Survival data was gathered through the examination of medical records and the implementation of telephone interviews. In this research, we established that progression-free survival (PFS) refers to the duration between the surgical procedure and the occurrence of cancer advancement. The duration of survival (OS) was determined as the period starting from the surgical procedure until the occurrence of death. Patients who did not encounter any advancement or demise throughout the research had their follow-up terminated on 31 August 2021, marking the final day of follow-up.

### Immunohistochemistry and multi-color immunofluorescence

For this research, the antibodies employed consisted of anti-B7H3 (14058S; Cell Signaling Technology, Danvers, MA, USA)and a rabbit mAb IgG Isotype Control (3900, Cell Signaling Technology, Danvers, MA, USA) was used as a parallel control antibody. The protocols were followed to conduct immunohistochemical staining of B7H3. The 4 μm sections underwent deparaffinization, rehydration, and incubation with EDTA (pH = 9.0). Subsequently, they were subjected to heating at 800 W for 2 minutes, followed by heating at 200 W for 10 minutes in a microwave oven (Midea, M1-211A, China). Next, the sections were blocked using 0.3% hydrogen peroxide for a duration of 10 minutes and 5% bovine serum albumin for 1 hour. Following that, they were treated with anti-B7H3 (1:100), and subsequently processed using biotinylated secondary antibodies. The slides were observed with Olympus IX-71 (Olympus, Japan).

To perform multi-color immunofluorescence staining, the TSAPLus Fluorescent Triple Staining Kit (G1236, Servicebio, Wuhan, China) was utilized according to the provided instructions. After overnight incubation with primary antibodies, the sections were subsequently treated with polymer HRP Mouse/Rabbit for 50 minutes and then incubated with tyramide for an additional 10 minutes. After being bound, the primary and secondary antibodies were eliminated by applying heat-induced epitope retrieval treatment with a stripping buffer. Following the rinsing of the specimen with chilled flowing faucet water and TBST (Tris-buffered saline with Tween), the procedure of staining and elimination of antibodies was repeated with an alternative iF-tyramide. This allows for the detection of multiple targets or markers in the sample using different fluorescence colors. In the end, the specimens were prepared by applying ProLong™ Diamond Antifade mountant with DAPI (G1236, Servicebio, Wuhan, China).

### Quantification of B7H3

The expression of B7H3 was assessed as either negative (no or weak staining) or positive (moderate to strong staining). In cases where different staining intensities were observed in two spots from the same case, a higher score was recorded. All slides underwent independent review by a minimum of two out of three observers who were unaware of the clinical outcomes. In the event of any discrepancies, a consensus was reached through discussion and examination under a microscope (Olympus IX-71, Olympus, Japan).

### Statistical analysis

The statistical analysis was performed utilizing the GraphPad Prism 8.0 (GraphPad, Bethesda, MD). Associations between the expression of B7H3 and clinicopathological characteristics were assessed using the chi-square test or Fisher’s exact test and presented in a cross-table format. Two-group analysis was conducted using the student’s *t*-test. Survival curves for PFS and OS were generated using the Kaplan-Meier method, and intergroup differences in survival were evaluated using the log-rank test.

## Results

### Identification of cell subpopulations in scRNA-seq samples

**Figure 1:**
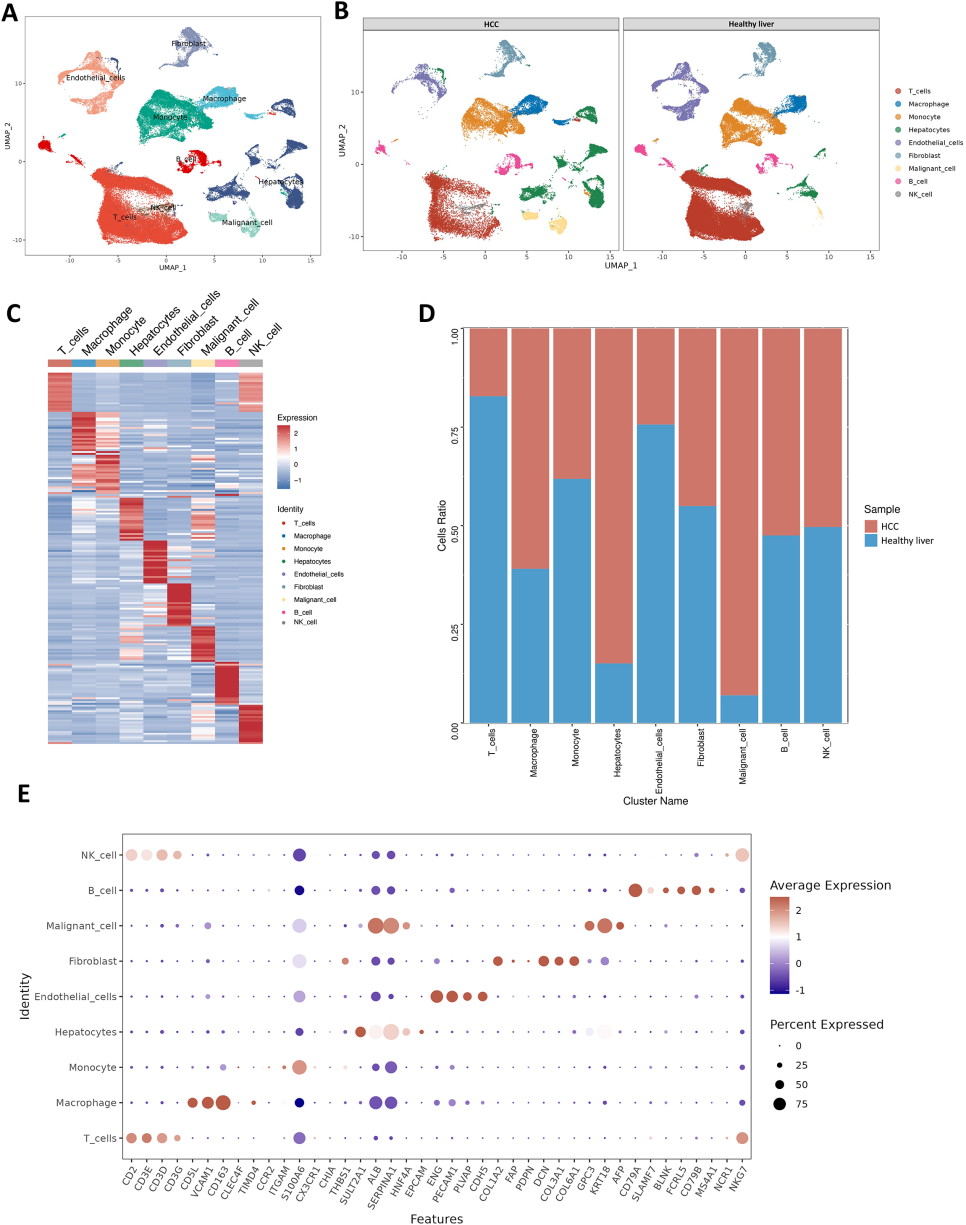
Healthy liver and tumor samples. **(A)** t-SNE plot of 42 samples. **(B)** t-SNE plot comparing healthy liver and liver cancer samples. **(C)** Heatmap showing the expression of marker genes. Red indicates higher expression levels, while blue indicates lower expression levels. **(D)** Bar graph showing the relative proportions of cell types in healthy liver and liver cancer. Each cell type is represented by a different color. **(E)** Bubble plot displaying the expression of marker genes in major cell types. The color of the dots reflects the expression level, and the size of the dots represents the percentage of cells expressing the marker gene in different cell types.

This study incorporated 42 single-cell samples for analysis, comprising 18 samples of HCC and 24 samples of healthy liver tissue. **Figure 1A** presents an overview of the single-cell data from both healthy and tumor samples. **Figure 1B** provides a summary of single-cell data across different groups. Visualization of t-SNE clustering revealed 89,246 cells from HCC and normal tissues clustered into 37 (0-36) subgroups. We annotated the expression of marker genes for each cluster (**Figure 1C**). The cell ratios of HCC samples and healthy liver samples among different cell types are depicted in **Figure 1D**. To elucidate the characteristics of each cell subgroup, we analyzed the marker genes across all subgroups. Heatmaps and bubble plots of the top genes for all subgroups are displayed in **Figure 1E**. We identified eight cell types, including Hepatocytes (SULT2A1, ALB, SERPINA1, HNF4A), Malignant cells (Gpc3, KRT18, AFP), Monocytes (CCR2, ITGAM, S100A6), Macrophages (VCAM1, CD163, CLEC4F), NK cells (NCR1, NKG7), B cells (CD79A, SLAMF7, BLNK, FCRLS, CD79B, MS4A1), T cells (CD3D, CD3E, CD3G, CD2), Fibroblasts (COL1A2, FAP, PDPN, DCN, COL3A1, COL6A1) and Endothelial cells (CDH5, PEPCAM1, ENG, PLVAP).

### Distribution of the B7/CD28 family across different subpopulations

**Figure 2:**
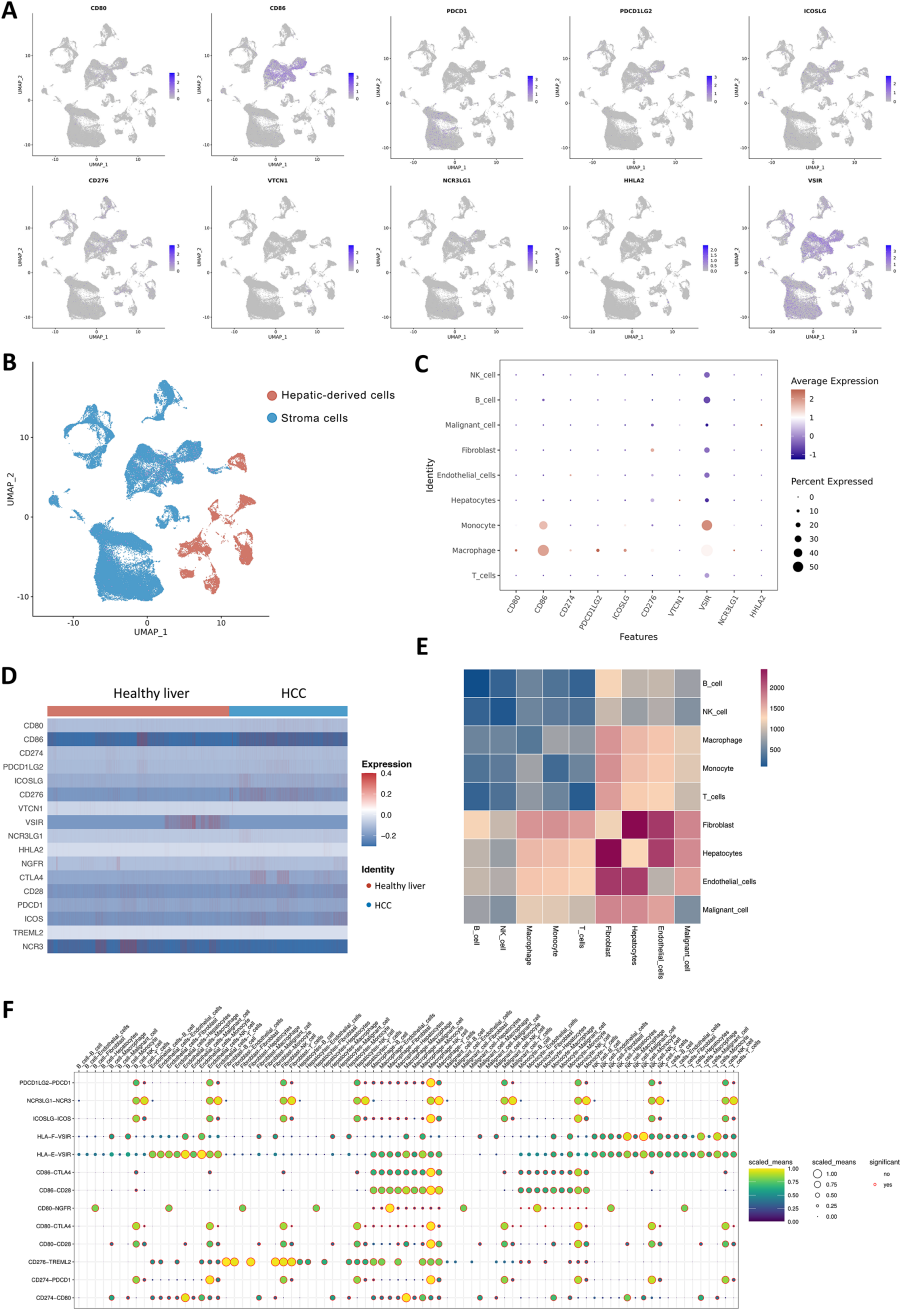
Cell-Cell Ligand-Receptor Interactions Predicted by CellChat in B7/CD28 family Protein Tumor Cells, Endothelial Cells, and Immune Cells. **(A)** The expression of B7/CD28 family proteins in the clusters. **(A, B)** t-SNE plot of hepatic-derived cells and stroma cells. **(C)** Bubble plots of the B7/CD28 family protein in different cell types. **(D)** Heatmap showing the B7/CD28 family gene expressions across the 11 cell types in healthy liver and healthy Liver and tumor Samples. **(E)** Bubble plot of ligand-receptor pairs for B7/CD28 family proteins. The color of the dots reflects the communication probability, and the size of the dots represents the computed p-value. Each cell group is displayed using a different color. Blank space indicates a communication probability of zero. The p-values are calculated using a two-sided permutation test. **(F)** Heatmap showing the strength of interactions between healthy liver and liver tumor cells.

Initially, we investigated the distribution of the B7/CD28 family among different cell types. The B7/CD28 family protein is mostly expressed in macrophages, monocytes, T cells, hepatocytes, malignant cells, and endothelial cells (**Figures 2A–C**). **Figure 2B** shows the distribution of hepatic-derived cells (hepatocytes and malignant cells) and stroma cells (including fibroblasts, endothelial cells, macrophages, and other cells), where the red color represents Hepatic-derived cells and the blue color represents Stroma cells. We investigated the differential expression of the B7/CD28 family between healthy liver tissue and HCC samples, noting an elevation in the expression of CD80, CD86, PDL1, HHLA2, NCR3LG1, CD276 and a decrease in the expression of CD274, VTCN1, PDCD1, VSIR in HCC samples (**Figure 2D**). **Table 1** summarizes the receptor-ligand relationships of the B7/CD28 family and their distribution across various cell types.

**Table 1:** B7 family ligand and the ligand alias.

B7 family ligand	Ligand alias	Main Expression	Putative receptors	Cell-cell communication
B7-1	CD80	Macrophage/monocyte	NGFR	B cell, endothelial cell, fibroblast, hepatocyte, malignant cell, macrophage, monocyte, NK cells, T cell - Fibroblast
			CTLA-4	B cell, endothelial cell, fibroblast, hepatocyte, malignant cell, macrophage, monocyte, NK cells, T cell - NK cell; Macrphage - B cell, endothelial cell, fibroblast, hepatocyte, malignant cell, macrophage, monocyte, NK cells, T cell;
			CD28	B cell, endothelial cell, fibroblast, hepatocyte, malignant cell, macrophage, monocyte, NK cells, T cell - NK cell/T cell/Macrophage
B7-2	CD86	Macrophage/monocyte	CTLA-4	Macrophage/monocyte - B cell, endothelial cell, fibroblast, hepatocyte, malignant cell, macrophage, monocyte, NK cells, T cell; B cell, endothelial cell, fibroblast, hepatocyte, malignant cell, macrophage, monocyte, NK cells, T cell - NK cell
			CD28	Macrophage/monocyte - B cell, endothelial cell, fibroblast, hepatocyte, malignant cell, macrophage, monocyte, NK cells, T cell
B7-H1	PD-L1	Macrophage/monocyte/endothelial cell	PDCD1	NK cell/T cell - B cell, endothelial cell, fibroblast, hepatocyte, malignant cell, macrophage, monocyte, NK cells, T cell
			CD80	Endothelial cell/Macrophage - B cell, endothelial cell, fibroblast, hepatocyte, malignant cell, macrophage, monocyte, NK cells, T cell; B cell, endothelial cell, fibroblast, hepatocyte, malignant cell, macrophage, monocyte, NK cells, T cell - macrophage/monocyte.
B7-DC	PD-L2	Macrophage/monocyte/endothelial cell	PDCD1	Macrophage/monocyte - B cell, endothelial cell, fibroblast, hepatocyte, malignant cell, macrophage, monocyte, NK cells, T cell; B cell, endothelial cell, fibroblast, hepatocyte, malignant cell, macrophage, monocyte, NK cells, T cell - NK cell/T cell
B7-H2	ICOSLG	Macrophage/monocyte/B cell	ICOS	Macrophage/monocyte - B cell, endothelial cell, fibroblast, hepatocyte, malignant cell, macrophage, monocyte, NK cells, T cell; B cell, endothelial cell, fibroblast, hepatocyte, malignant cell, macrophage, monocyte, NK cells, T cell - NK cell/T cell
B7H3	CD276	Hepatocyte/macrophage/monocyte/endothelial cells/Fibroblast	TREML2	Hepatocyte/macrophage/monocyte/endothelial cells/Fibroblast - B cell, endothelial cell, hepatocyte, monocyte, NK cells, T cell
B7-H4	VTCN1	Hepatocyte/malignant cell	NA	NA
B7-H5	VSIR	Macrophage/monocyte/T cell	HLA-F	Macrophage/monocyte - B cell, endothelial cell, fibroblast, hepatocyte, malignant cell, macrophage, monocyte, NK cells, T cell
			HLA-E	B cell, endothelial cell, fibroblast, hepatocyte, malignant cell, macrophage, monocyte, NK cells, T cell - Endothelial cell/macrophage/NK cell/T cell
B7-H6	NCR3LG1	Macrophage/monocyte/T cell	NCR3	NK cell/T cell
B7-H7	HHLA2	Malignant cell	NA	B cell, endothelial cell, fibroblast, hepatocyte, malignant cell, macrophage, monocyte, NK cells, T cell - NK cell/T cell

### Cell-cell interaction analysis related to the B7/CD28 family in scRNA-seq

We analyzed the number and weight of ligand-receptor pairs. Macrophages, endothelial cells, tumor cells, fibroblasts, hepatocytes, and NK cells exhibited relatively active cell communication in healthy liver tissue among all cell types. Similarly, in liver tumor tissue, macrophages, endothelial cells, tumor cells, fibroblasts, hepatocytes, and NK cells displayed relatively active cell communication among all cell types (**Figure 2E**).

To explore the role of the B7/CD28 family proteins within the TME, we conducted an intercellular ligand-receptor analysis specific to the B7/CD28 family proteins. We compiled the receptor-ligand relationships of the B7/CD28 family from the latest version of CellPhoneDB, and the summary is presented in the provided **Table 1**. Initially, we summarized the receptor-ligand relationships of the B7/CD28 family (**Table 1**). Among them, interactions mediated by PDCD1LG2-PDCD1, NCR3LG1-NCR3, ICOSLG-ICOS, HLA-F-VSIR, HLA-E-VSIR, CD86-CTLA4, CD86-CD28, CD80-NGFR, CD80-CTLA4, CD80-CD28, CD276-TREML2, CD274-PDCD1, CD274-CD80 were observed between tumor cells and stromal cells. Notably, there was strong cell communication between hepatocytes, endothelial cells, and fibroblasts (**Figure 2F**).

### Establishing and validating a signature for the prognosis of B7/CD28 family-associated proteins

To further investigate the prognostic roles of proteins and receptors in the B7/CD28 family in HCC, transcriptomic profiles and clinical data were acquired from the TCGA portal for a total of 377 individuals diagnosed with liver hepatocellular carcinoma (LIHC). 365 patients were chosen for further analysis after excluding patients with a follow-up period shorter than 30 days.

**Figure 3:**
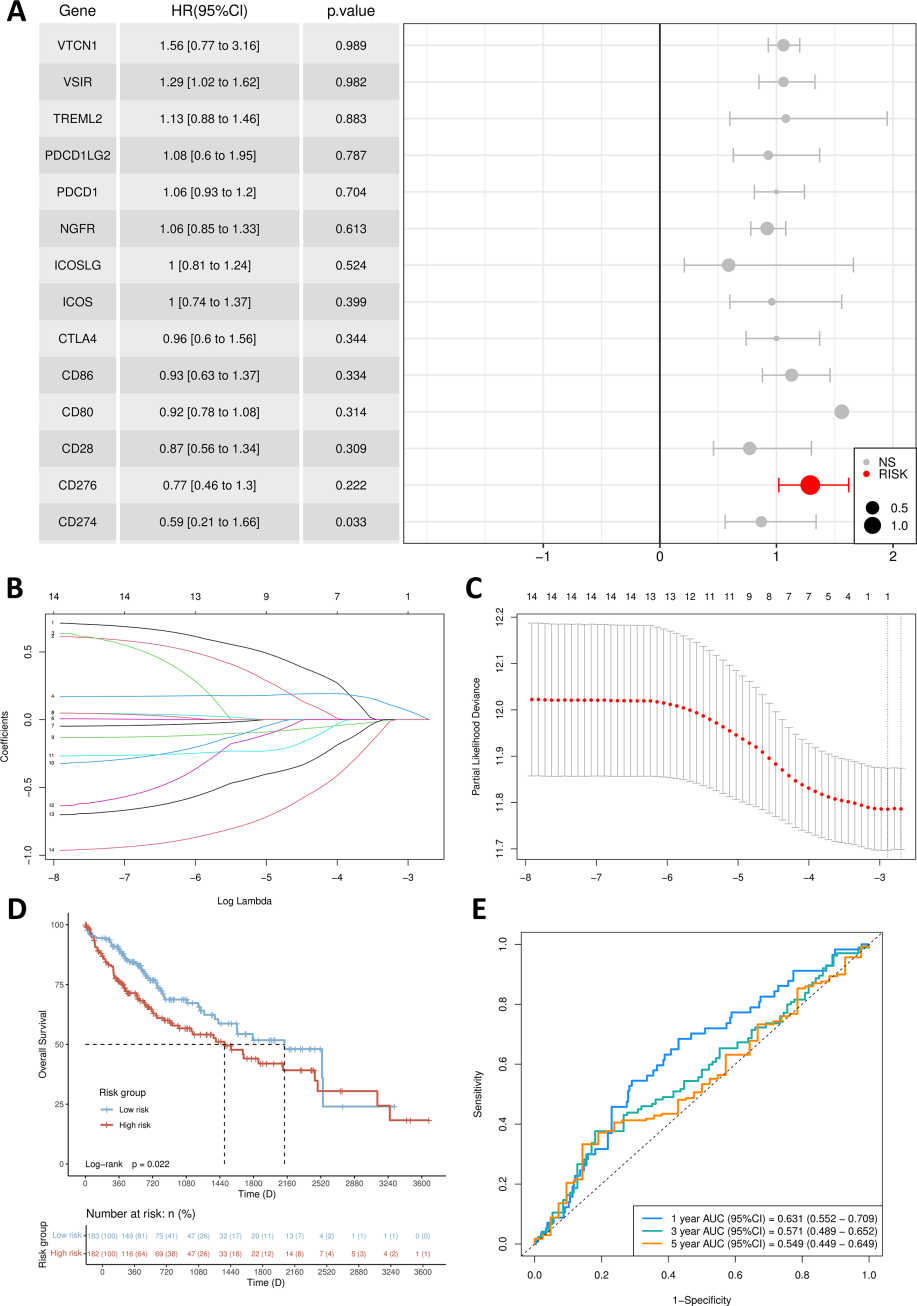
Establishment of prognosis signature. **(A)** Multivariate Cox regression forest plot. **(B)** B7/CD28 family proteins were analyzed by LASSO regression. **(C)** The tuning parameter selection of the LASSO analysis. **(D)** Kaplan-Meier survival curves for the high-risk group and low-risk group. **(E)** The AUCs for 1-, 3- and 5-year ROC curves.

After conducting a single-factor Cox regression analysis (**Figure 3A**), it was found that only one gene, B7H3 (CD276), among the proteins and receptors of the B7/CD28 family could potentially be linked to prognosis. Following the implementation of multivariate Cox regression analysis, the genes and their respective regression coefficients were employed to construct a prognostic signature (**Figure 3B**).

Through the application of the lasso algorithm, B7H3 was identified as the sole risk factor. Subsequently, a predictive model was established. We utilized the median value of the calculated risk scores as a cut-off point to categorize the 365 samples into high-risk and low-risk groups. PCA analysis vividly illustrated that patients were clearly separated into two distinct clusters based on their risk levels (**Figure 3C**). Evidently, compared with the low-risk group, the high-risk group had a higher fatality rate and a shorter lifespan. By performing Kaplan-Meier survival analysis and the log-rank test, we determined that the low-risk group had a significantly longer survival duration (*p* = 0.0222) (**Figure 3D**). As depicted in **Figure 3E**, in the time-dependent ROC curve analysis, the prognostic signature exhibited an AUC value of 0.631 for 1-year survival, 0.571 for 3-year survival, and 0.549 for 5-year survival.

### Patient clinicopathological characteristics

After the preliminary discovery that B7H3 plays a role in prognosis, we retrospectively collected patients diagnosed with liver cancer in our hospital to analyze the distribution of B7H3 in liver cancer and the differences in its role in prognosis. A total of 94 participants were enrolled in the research, and their initial clinicopathological traits are provided in **Table 2**. The median age of the participants was 55 years, ranging from 25 to 78 years. Among these participants, 21 cases of HCC were of the diffuse type, while the remaining 73 cases were of the well - circumscribed type. After the monitoring period, HCC resulted in the demise of 71.28% (67 out of 94) of the patients. The median duration of OS was 21–40 months, with a range of 1 to 86 months. Out of a total of 94 patients, disease progression was observed in 82.98% (78 patients). The median time for PFS was 30.5 months, with a range of 1 to 85 months.

**Table 2:** Clinical and biological features of clinical HCC patients.

Variables	PatientsNO.	B7H3 expression		
		Low	High	*p*
Age(y)				
<55	38	8	30	0.0953
≥55	56	4	52	
Gender				
Male	84	12	72	0.3509
Female	10	0	10	
Alcohol				
+	13	1	12	0.8864
–	81	11	70	
Cirrhosis				
+	42	7	35	0.3084
–	52	5	47	
HBV				
+	70	10	60	0.6894
–	24	2	22	
Tumor size				
>50 mm^3^	50	4	46	0.2435
≤50 mm^3^	44	8	36	
Tumor nodules				
Single	83	10	73	0.9266
Multi	11	2	9	
Satellite nodules				
+	8	0	8	0.5902
–	86	12	74	
vascular invasion				
+	6	0	6	0.9999
–	88	12	76	
AFP serum level				
>300 ng/ml	26	4	24	0.9599
≤300 ng/ml	66	8	58	

### Expression of B7H3 in HCC

**Figure 4:**
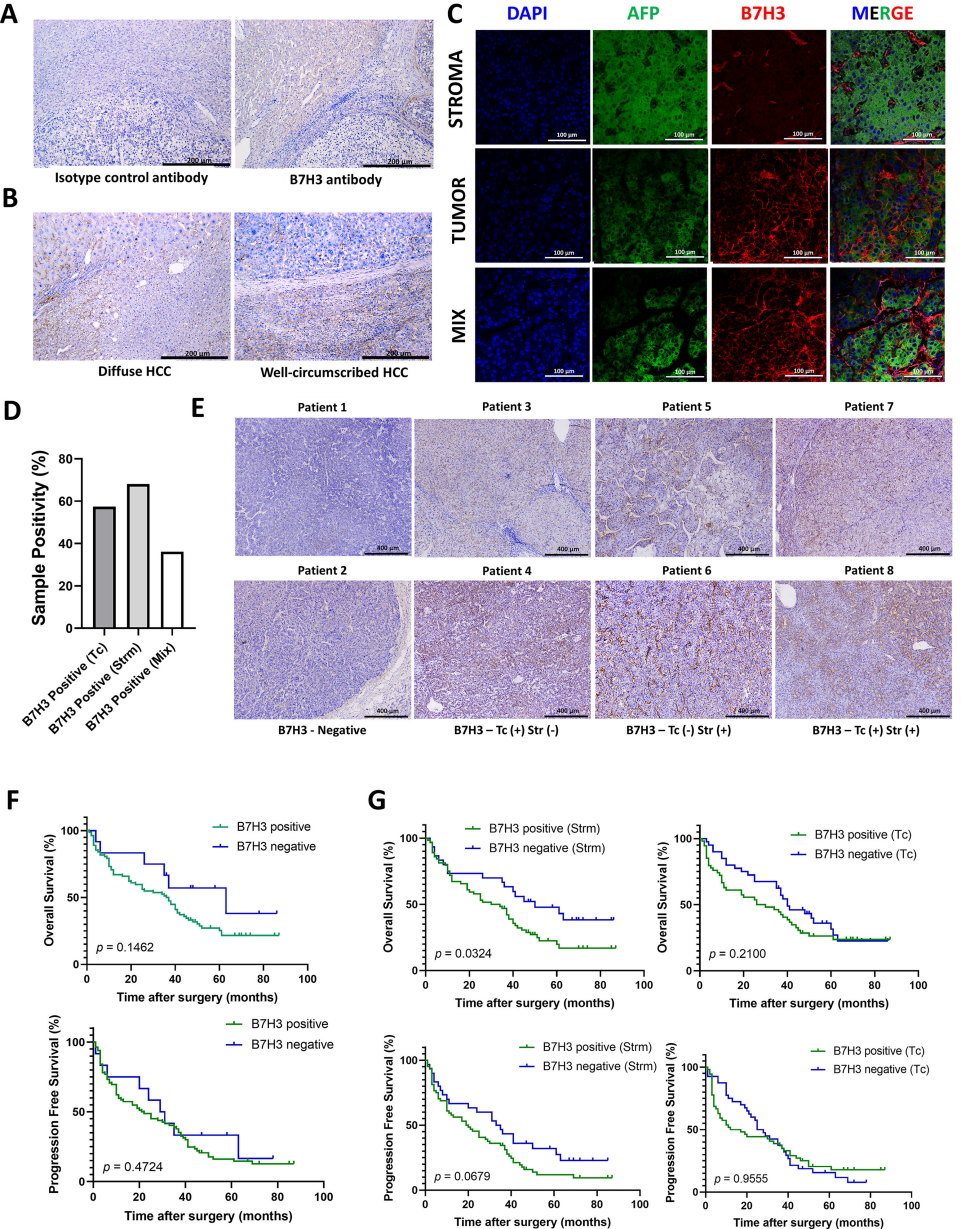
The expression pattern of B7H3 in tumor tissues. **(A)** The representative IHC images of Isotype control antibody and B7H3 antibody; **(B)** The representative IHC images of diffuse HCC and well-circumscribed HCC; **(C)** Representative multi-immunofluorescence images of AFP (green) and B7H3 (red). The nucleus is labeled with DAPI (blue). **(D)** Proportions of B7H3 on tumor cells (TCs), in stroma and in both. **(E)** The representative IHC images of B7H3 from serial sections. **(F)** OS and PFS for HCC patients based on the expression of B7H3 on tumor tissue. **(G)** OS and PFS for HCC patients based on the expression of B7H3 in the stroma and on tumor cells.

To examine the expression features of B7H3 in liver tumors, an analysis was conducted using IHC and IF techniques. To ensure the reliability of the results, the staining results were compared with isotype controls. The anti-B7H3 antibody showed a high degree of specificity (**Figure 4A**). We have found that B7H3 was expressed in 90% of liver tumors, aligning with earlier studies (14). Specifically, among the 21 diffuse - type HCC cases, 19 were B7H3 - positive, with a positive rate of approximately 90%; in the 73 well - circumscribed - type HCC cases, 66 were B7H3 - positive, also with a positive rate of about 90% (**Figure 4B**).

Multicolor immunofluorescence revealed that B7H3 displayed three distinct localization patterns: on tumor cells (Tc), in the stroma (Strm), and on both cell types concurrently (the mixed pattern) (**Figure 4C**). This finding is consistent with a prior single - cell RNA sequencing (sc - RNAseq) study, which reported that B7H3 was expressed on tumor cells/hepatocytes as well as stromal cells (including fibroblasts and macrophages). In this study, approximately 68% of patients showed B7H3 expression in the stroma, while 57.5% exhibited B7H3 expression on tumor cells. Around 36% of all patients had co - expression of B7H3 on both the stroma and tumor cells (**Figure 4D**). We included representative images from different patients, showing different expression levels and patterns of B7H3 (**Figure 4E**).

There were no statistically significant differences in the prognosis of liver cancer, in terms of OS (*p* = 0.1462) and PFS (*p* = 0.4742), between the groups exhibiting high and low expression of B7H3 (**Figure 4F**). Previous literature has indicated that the presence of B7/CD28 family molecules in different tumor components is associated with diverse prognoses (15). Our research found that the presence of B7H3 in stromal cells can accurately predict the prognosis of liver cancer (*p* = 0.0324 for OS and *p* = 0.0679 for PFS), while the presence of B7H3 on tumor cells had no impact on the prognosis of liver cancer (*p* = 0.2100 for OS and *p* = 0.9555 for PFS) (**Figure 4G**).

## Discussion

The B7/CD28 family of immune checkpoints stands as a pivotal family governing the tumor immune microenvironment. Particularly, PD-1 and CTLA4 inhibitors have been applied in clinical settings, significantly promoting the prognosis of cancer patients (16). Researchers are progressively delving deeper and elucidating further members within this family (17–19). In this study, we consolidated single-cell sequencing findings from 4 databases regarding healthy liver and liver cancer, methodically examining the expression levels, cell types, and intercellular communications of the B7/CD28 family within healthy liver and liver cancer. According to our study, the B7/CD28 family showed active cell-cell communications among malignant cell, macrophages, endothelial cells, and so on, indicating the active role played by the B7/CD28 family in the immune microenvironment.

Secondly, according to the data obtained from TCGA, LASSO was employed in this investigation to examine the prognostic significance of the B7/CD28 family in individuals with liver cancer. The study uncovered a connection between unfavorable prognosis and elevated levels of B7H3. In our hospital, we additionally recruited a cohort of individuals diagnosed with liver cancer and discovered that the rate of B7H3 positivity in liver cancer tissue was 90%. Through the utilization of multi-color immunofluorescence, we made an intriguing revelation regarding the localization patterns of B7H3 in liver cancer tissue. These patterns encompass the presence of these proteins on tumor cells, stroma, and a combination of both (mix pattern). Additional examination of the prognosis of individuals with liver cancer indicated that the primary factor contributing to an unfavorable prognosis was the presence of B7H3 in stroma.

B7 homolog 3 (B7H3), also known as CD276, is highly overexpressed in tumor cells, related endothelial cells, cancer - associated fibroblasts (CAFs), macrophages, etc., but less so in normal tissues. In the tumor microenvironment, B7H3 crucially regulates immune cells, CAFs, and endothelial cells, contributing to a tumor - promoting environment (20). This environment facilitates uncontrolled cancer cell proliferation, increased metabolism, growth of cancer stem cells, and resistance to standard treatments (21). Blocking B7H3 and ending its immunosuppressive functions may enhance the anti - tumor immune response and impede tumor progression (22). In our cell - communication analysis, we found that CD276 and its receptors have extensive connections among hepatocytes, macrophages, monocytes, endothelial cells, fibroblasts, as well as B cells, endothelial cells, hepatocytes, monocytes, NK cells, and T cells. This also suggests that it may be related to angiogenesis and activities within the immune environment.

It has been provided that B7H3 expressed on cancer cells acts as a significant impediment to T cell growth and stimulation in HCC (21). Moreover, preclinical studies have indicated a promising therapeutic strategy: targeting B7H3 present not only on tumor cells but also on vascular surfaces (23). This approach has been shown to effectively treat primary and metastatic tumors in mouse models, offering new insights into potential cancer therapies. In addition, the expression of B7H3 on CAFs has been identified as a predictor of poor prognosis (24). In ovarian cancer, the survival rate can be predicted by the expression of B7H3 in tumor-associated endothelial cells (15). A study has shown that the expression of B7H3 in tumor cell is associated with the prognosis of HCC patients (21). While our results showed that the expression in the stroma had a closer relationship with poor prognosis. In our study, B7H3 in the tumor stroma may have led to poor prognosis by regulating the stromal microenvironment and influencing immune cell activities and angiogenesis.

Our study had several advantages: Primarily, through single-cell sequencing, we have gained a comprehensive understanding of the expression and distribution of the B7/CD28 family in liver cancer. Secondly, we utilized bioinformatics to address the most significant compounds within the B7/CD28 family for predicting liver cancer outcomes. Furthermore, we employed a multi-color immunofluorescence technique to examine the expression patterns of the most significant molecules for prognosis. This approach enhances the precision of our prediction model and provides unique insights not accessible through current bioinformatics methods.

In terms of treatment, this study underlined the potential role of B7-H3 in the prognosis analysis and targeted therapy of liver cancer. However, our study included samples with a certain degree of overlap (samples with B7H3 expression in both tumor cells and stromal cells), and these samples were included in both the tumor cell group and the stromal cell group to comprehensively evaluate the role of B7H3 in the prognosis of HCC patients. Although this grouping method allows for a more comprehensive analysis of B7H3. Larger - scale and in - depth studies are still necessary. Another notable limitation is its retrospective design, which solely relies on data collected from a single center, which inevitably introduces inherent biases. Also, due to our sample size, we were unable to perform subgroup analyses of different pathological types of liver cancer. Similarly, we need more and larger studies to verify our results. Further investigation in cell biology is still needed to better understand the association of B7H3 with prognosis in liver cancer patients.

## Conclusion

In conclusion, this study has shed light on the critical role of the B7/CD28 immune checkpoint family, particularly B7H3, in the TME of HCC. Analysis of single-cell sequencing and TCGA data has revealed a correlation between high B7H3 expression and poor prognosis. Advanced multi-color immunofluorescence techniques have detailed the distribution of B7H3 in endothelial cells, associating its expression with unfavorable patient outcomes. Although preclinical models suggest the potential of B7H3 as a target for treatment, its application in HCC treatment requires validation through more extensive clinical trials. Given the retrospective design and the limitation of being a single-centric study, broader multicentric research is needed. Overall, our research opens avenues for the clinical implementation of B7H3 as a prognostic marker and therapeutic target in liver cancer.

## Data Availability

The original contributions presented in the study are included in the article/supplementary material. Further inquiries can be directed to the corresponding author.
